# Elevated intracellular copper contributes a unique role to kidney fibrosis by lysyl oxidase mediated matrix crosslinking

**DOI:** 10.1038/s41419-020-2404-5

**Published:** 2020-03-31

**Authors:** Yang-yang Niu, Ying-ying Zhang, Zhi Zhu, Xiao-qin Zhang, Xi Liu, Sai-ya Zhu, Ye Song, Xian Jin, Bengt Lindholm, Chen Yu

**Affiliations:** 1 Department of Nephrology, Shanghai Tongji Hospital, Tongji University School of Medicine, Shanghai, China; 2 Terahertz Technology Innovatio, Research Institute, Shanghai Key Lab of Modern Optical System, Terahertz, Science Cooperative Innovation Center, School of Optical-Electrical Computer, Engineering, University of Shanghai for Science and Technology, Shanghai, China; 3 Department of Ultrasound, Zhoupu Hospital, Shanghai University of Medicine & Health Sciences, Shanghai, China; 4 EnnovaBio Pharmaceuticals Co., Ltd, Shanghai, China; 5 Division of Renal Medicine and Baxter Novum, Department of Clinical Science, Intervention and Technology, Karolinska Institutet, Stockholm, Sweden

**Keywords:** Metals, Kidney, Chronic kidney disease

## Abstract

Copper ions play various roles in mammalian cells, presumably due to their involvement in different enzymatic reactions. Some studies indicated that serum copper correlates with fibrosis in organs, such as liver and lung. However, the mechanism is unknown. Here, we explored the role of copper in kidney fibrosis development and possible underlying mechanisms. We found that copper transporter 1 (CTR1) expression was increased in the kidney tissues in two fibrosis models and in patients with kidney fibrosis. Similar results were also found in renal tubular epithelial cells and fibroblast cells treated with transforming growth factor beta (TGF-β). Mechanistically, the upregulation of CTR1 required Smads-dependent TGF-β signaling pathway and Smad3 directly binded to the promoter of CTR1 in renal fibroblast cells using chromatin immunoprecipitation. Elevated CTR1 induced increase of copper intracellular influx. The elevated intracellular copper ions activated lysyl oxidase (LOX) to enhance the crosslinking of collagen and elastin, which then promoted kidney fibrosis. Reducing intracellular copper accumulation by knocking down CTR1 ameliorated kidney fibrosis in unilateral ureteral obstruction induced renal fibrosis model and renal fibroblast cells stimulated by TGF-β. Treatment with copper chelator tetrathiomolybdate (TM) also alleviated renal fibrosis in vivo and in vitro. In conclusion, intracellular copper accumulation plays a unique role to kidney fibrosis by activating LOX mediated collagen and elastin crosslinking. Inhibition of intracellular copper overload may be a potential portal to alleviate kidney fibrosis.

## Introduction

Kidney fibrosis is the principal process and the final common pathway underlying the progression of all chronic kidney disease (CKD) to end stage of kidney disease (ESKD). Kidney fibrosis is characterized by excess accumulation of extracellular matrix (ECM) substances in kidney. Because there are currently no specific treatments for renal fibrosis, a deeper understanding of the molecular and cellular basis of renal fibrosis will be beneficial to the development of effective strategies that diminish or even reverse kidney fibrosis in CKD.

Copper is one of the most important trace elements in the human body, as it is a cofactor or structural component for many enzymes that are required for cellular physiology^1,2^. Recently, some studies have shown that copper accumulation is associated with fibrosis in various tissues such as in human liver^3^, oral submucous tissue in humans^4^ and lung fibrosis in rats^5^. However, there have been no reports of association of copper with fibrosis in the kidney, and the underlying mechanism involving the role of copper in organ fibrosis is unknown.

The core foundation of fibrosis is the ECM, which includes elastin, collagen, fibronectin, and oxytalan fibers. Under physiological conditions, most of these molecules are assembled into microfibrils, which are arranged in parallel in quarter-staggered arrays with overlap and gap regions, assembled into three-dimensional fibrils, and finally, the elastin and collagen molecules become insoluble. This process is called ECM crosslinking and is critical for the maintenance of a stable matrix^6,7^.

Lysyl oxidase (LOX), a copper-dependent enzyme, is the most important enzyme for ECM crosslinking. LOX becomes active when combined with copper in the Golgi and is then secreted extracellularly to crosslink elastin and collagen^8,9^. Therefore, the level of intracellular copper determines the activity of LOX.

Under pathological conditions, overproduction ECM and overactivated ECM crosslinking contributes to organ fibrosis^10^. To date, most studies on organ fibrosis have focused on the accumulation of ECM; however, studies targeting ECM crosslinking for fibrosis are rare.

In this study, we found that the levels of intracellular copper were significantly elevated in two kidney fibrosis models. We also found that the expression of copper transporter 1 (CTR1), the major transporter for copper influx^11^, was significantly increased in the fibrotic kidney. We revealed that the activities of LOX were increased in the fibrotic kidneys and were accompanied by increased ECM crosslinking. Either CTR1 downregulation or copper chelation could alleviate renal fibrosis. The same results were achieved in vitro. In conclusion, we identified a copper-dependent novel mechanism underlying kidney fibrosis in which upregulation of CTR1 leads to increased copper influx, which then increases LOX activity and thus promotes ECM crosslinking and fibrosis.

## Results

### The copper level is elevated in kidney fibrotic tissue

**Fig. 1:**
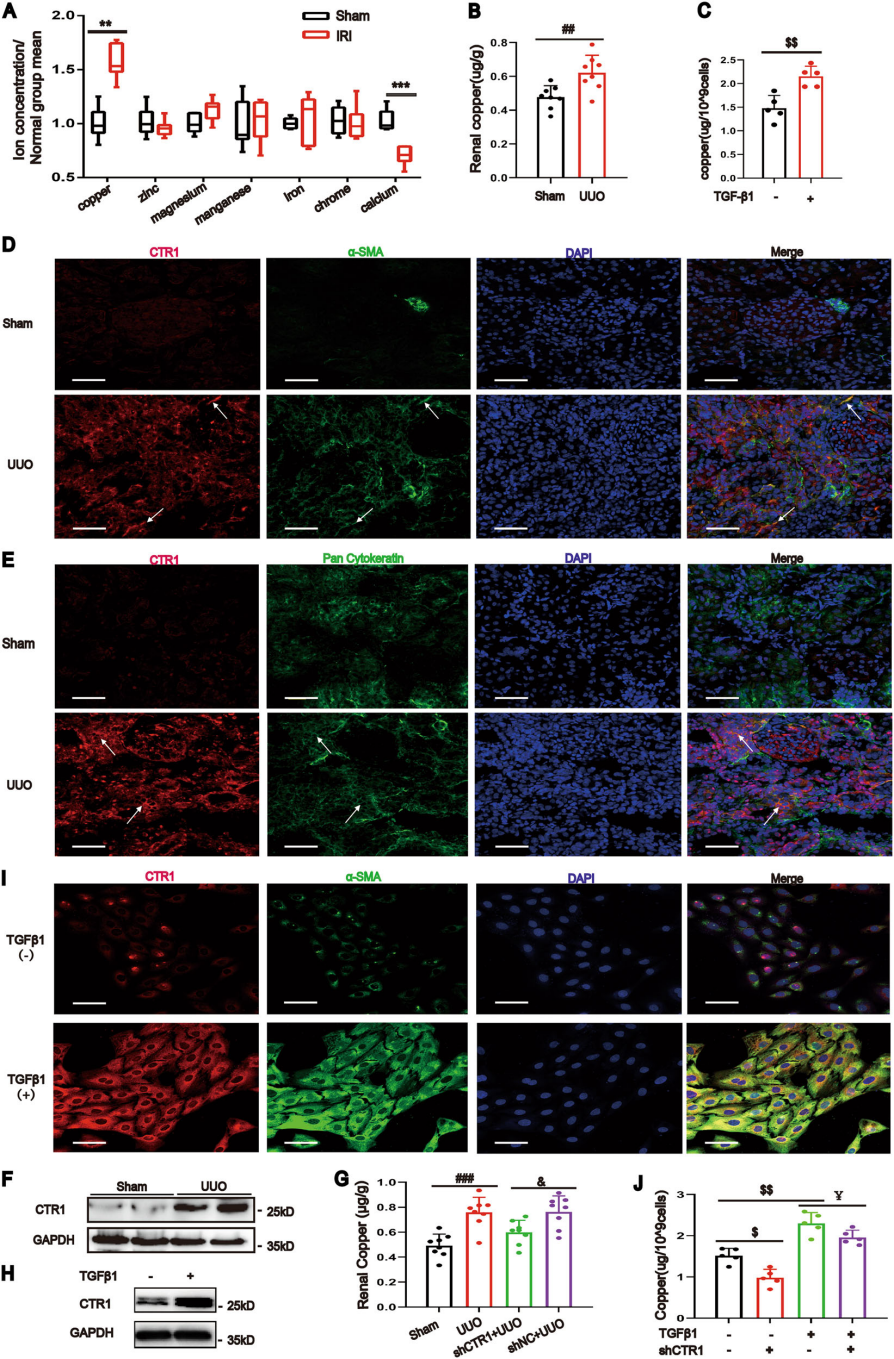
Renal copper and CTR1 are markedly increased in renal fibrosis. **a** Kidney copper, zinc, magnesium, manganese, iron, chrome and calcium concentrations were determined by ICP-MS analysis on day 28 after uIRIx operation (*n* = 6). **b** ICP-MS analysis of copper concentrations in sham and UUO kidneys on day 7 (*n* = 8). **c** ICP-MS analysis showing the copper concentrations in NRK-49F cells after 48 h of TGF-β1treatment (*n* = 5). **d** Immunofluorescent co-staining on sham and UUO kidney sections showed that CTR1 and α-SMA were increased in UUO kidney sections (*n* = 3). Bar = 50 μm. **e** Rat kidney sections were stained with CTR1 and Pan Cytokeratin antibody (*n* = 3). Bar = 50 μm. **f** Representative western blotting analysis showing the expression of CTR1 in sham and UUO kidneys on day 7 (*n* = 8). **g** The CTR1 shRNA–expressing plasmid or the empty plasmid mixed with lipid microbubble were delivered into the rat kidneys immediately after the UUO operation through the ultrasound-mediated gene transfer technique. Renal copper concentrations were tested by ICP-MS (*n* = 8). **h** Western blotting showing CTR1 upregulation in NRK-49F cells treated with TGF-β1 (*n* = 3). **i** CTR1 and α-SMA were analyzed by immunofluorescent co-staining in NRK-49F cells with or without TGF-β1 treatment (*n* = 3). Bar = 50 μm. **j** ICP-MS was used to analyze the copper concentrations in NRK-49F cells after CTR1 downregulation by lentivirus (*n* = 5). Data represent the mean ± SEM. **P < 0.01, ***P < 0.001 versus sham-operated mice. ##*P* < 0.01, ###*P* < 0.001 versus sham-operated rats. $$*P* < 0.01 versus the nontreated group. &*P* < 0.05 versus with the scramble-treated UUO kidneys. ¥*P* < 0.05 versus TGF-β1-treated cells.

We assessed the levels of some metal ions in the kidney tissue of the uIRIx (unilateral kidney ischemia/reperfusion injury with contralateral nephrectomy, sacrificed at 28 days) induced renal fibrosis model and found that only copper level was significantly increased, but not other metal ions, such as zinc, magnesium, manganese, iron, chrome, and calcium, determined by inductively coupled plasma-mass spectrometry (ICP-MS), compared to that in the sham mice (Fig. 1a). Similarly, kidney copper was also significantly elevated in the unilateral ureteral obstruction (UUO) induced renal fibrosis model (Fig. 1b). We also found that the copper level was upregulated in the normal rat kidney fibroblastic cell line (NRK-49F) and the normal rat kidney proximal tubular cells (NRK-52E) when treated with TGF-β1, the major mediator of renal fibrosis^12,13^ (Fig. 1c and Supplementary Fig. 1A).

### Elevated copper is due to enhanced CTR1

CTR1 is the major transporter for copper uptake in eukaryotes. By double immunofluorescence staining, we found that CTR1 was in renal interstitial myofibroblast cell (costaining with α-SMA) (Fig. 1d) and was also discovered in the tubular epithelial cell (costaining with Pan cytokeratin) (Fig. 1e). The expressions of CTR1 was obviously upregulated in the kidney of UUO model, compared to that in the sham group (Fig. 1d–f). When CTR1 was conditionally knocked down by small hairpin RNA (shRNA) (Supplementary Fig. 1C), the copper levels in the kidney tissue were significantly reduced compared with that in empty vector-treated UUO kidneys (Fig. 1g).

CTR1 expressions were upregulated after TGF-β1 treating in NRK-49F, NRK-52E and human proximal tubular epithelial (Fig. 1h, Supplementary Fig. 1D, E). CTR1 was co-localized with α-SMA in NRK-49F cells after TGF-β1 treatment by Immunofluorescence staining (Fig. 1i). When CTR1 was knocked down, the increasing of copper influx induced by TGF-β1 was blocked in NRK-49F cells (Fig. 1j and Supplementary Fig. 2A–C). Collectively, our results indicated that increasing CTR1 expression is responsible for increasing intracellular copper levels.

**Fig. 2:**
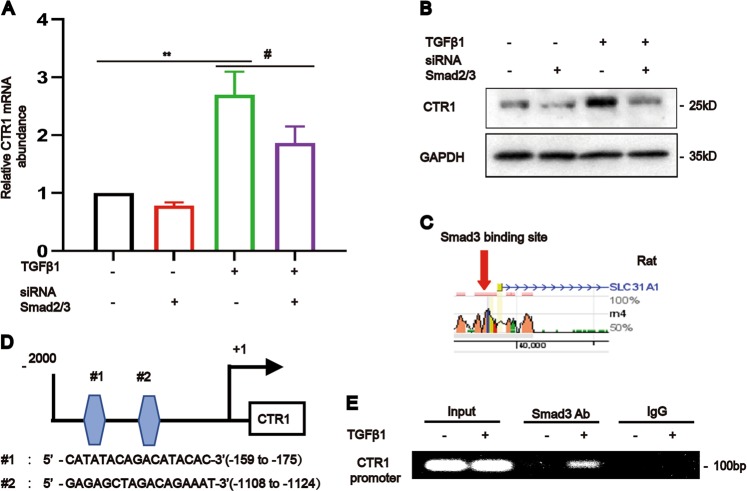
TGF-β1 upregulated CTR1 via Smad3 binding to the CTR1 promoter. **a**, **b** Real-time PCR and western blotting and analyses showed the expression of CTR1 in NRK-49F cells after the simultaneous knockdown of Smad2 and Smad3 and treatment as indicated (*n* = 5). **c**, **d** Binding protein prediction in the rat CTR1 promoters using two evolutionarily conserved predicted binding sites (http://ecrbrowser.dcode.org/ and http://jaspardev.genereg.net/) for Smad3. Note that the NRK-49F cell line used in this study originated in rats. **e** Chromatin immunoprecipitation (ChIP) assay followed by PCR showed that Smad3 physically binds to the CTR1 promoter in response to TGF-β1 after 24 h (*n* = 5). Data represent the mean ± SEM. **P < 0.01 versus blank control cells. #*P* < 0.05 versus TGF-β1-treated cells.

Then, we investigated the regulation of CTR1. The results showed that Smad2/3 knockdown inhibited the TGF-β1-induced enhancement of CTR1 mRNA and protein expression in NRK-49F cells (Fig. 2a, b). Further, by using transcription factor binding site prediction (http://www.rvista.dcode.org/ and http://jaspar.genereg.net/), we found two conserved Smad3-binding sites in the promoter region of the CTR1 (Slc31a1) gene (Fig. 2c, d)^14^. A Chromatin immunoprecipitation (ChIP) assay was used to clearly demonstrate the interaction between Smad3 and the CTR1 promoter (Fig. 2e). Therefore, CTR1 is a direct target gene of TGF-β1-Smad3 signaling.

### Elevated copper increases LOX activity to promote extracellular collagen cross-linking

**Fig. 3:**
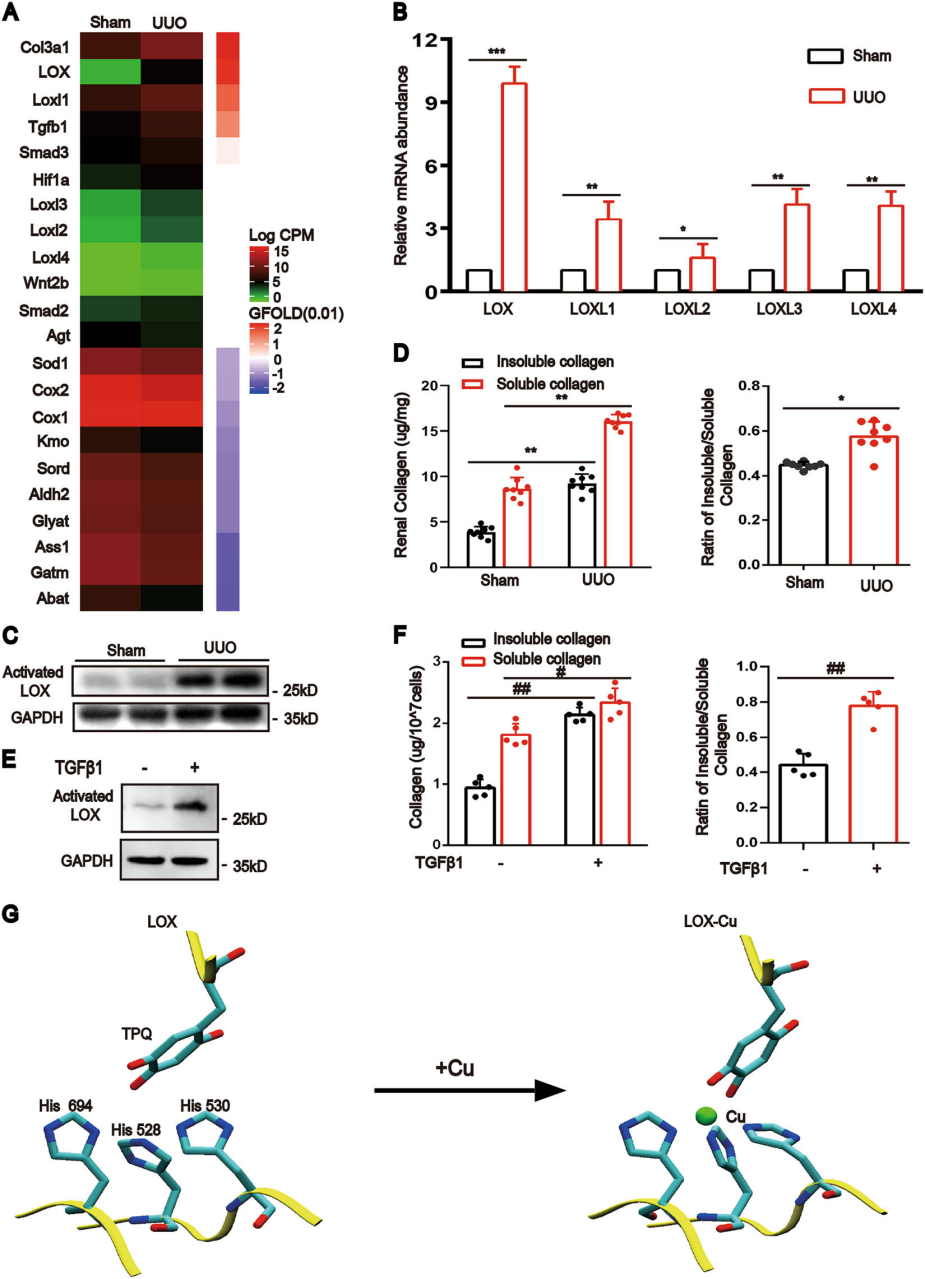
LOX activity is elevated during renal fibrosis development. **a** Heat map comparing the expression of copper-related genes and profibrotic genes in UUO kidneys with that in sham kidneys. **b** Real-time PCR measurement of the changes in mRNA levels of LOX, LOXL1, LOXL2, LOXL3, and LOXL4 (*n* = 3). **c** Western blotting showing the expression of activated LOX in UUO and sham kidneys (*n* = 5). **d** The absolute abundances of insoluble and soluble collagen in the rat kidneys were determined using the microwave-assisted acid digestion method and the ratio of insoluble to soluble collagen were assessed (*n* = 8). **e** Western blotting showing the expression of activated LOX in NRK-49F cells (*n* = 3). **f** Microwave-assisted acid digestion method showing the absolute abundances of insoluble and soluble collagen in NRK-49F cells and the ratio of insoluble to soluble collagen were calculated (*n* = 5). **g** Stereoview of activated LOX for saccharomycetes showing the ligands of copper. The binding energy of copper ions and LOX was calculated with the *ab initio* method based on density functional theory (DFT). Data represent the mean ± SEM. *P < 0.05, **P < 0.01, and ***P < 0.001 versus sham-operated rats. #*P* < 0.05, ##*P* < 0.01 versus the nontreated group.

To identify the precise target of copper, we performed transcriptome RNA sequence analysis of UUO kidneys. The results showed that some copper-dependent proteins were implicated. Notably, LOX and LOXL1–4 were significantly increased (Fig. 3a) in UUO kidneys compared with sham rats. Validating by real-time quantitative polymerase chain reaction (PCR), LOX, not LOXL1–4, was the most expressed profibrotic gene (Fig. 3b). The activated LOX protein was markedly increased (Fig. 3c). The insoluble collagen and the ratio of insoluble to soluble collagen were also significantly increased (Fig. 3d), which means that there was a marked increase of collagen cross-liking by LOX in the UUO kidneys when compared with that in the sham control kidneys. Similar results were observed in NRK-49F cells treated with TGF-β1 (Fig. 3e, f).

To determine the combining site of copper ions with LOX, we used saccharomycetes. The calculations using an ab initio method based on density functional theory (DFT) showed that His 528, His 530 and His 694 together with residue 478 (topaquinone) of LOX^15^(PDB ID: 1n9e) were the functional residues directly binding to copper ions (Fig. 3g). The charge analysis indicated that the Cu^2+^ ion was reduced to Cu^+^ after binding to LOX. To explore whether copper protects LOX from proteasomal degradation, we further calculated the binding energy (*E*_binding_) of the Cu ion and LOX by the formula *E*_binding_ = *E* (LOX-Cu^2+^) + 5*E* (H_2_O) – *E* ([Cu [H_2_O]_5_]^2+^) – *E* (LOX). The labels *E* (LOX-Cu^2+^), *E* (H_2_O), *E* ([Cu [H_2_O]_5_]^2+^), and *E* (LOX) represent the energies of the LOX-Cu complex, water molecule, hydrated Cu ion and LOX, respectively. The resulting binding energy was −149.2 kcal/mol, which was much stronger than the thermal fluctuation (~1 kcal/mol) of solution at room temperature, suggesting that the copper ion in solution can stably bind to LOX and further prevent decomposition of LOX.

### Knockdown of CTR1 improves renal fibrosis

**Fig. 4:**
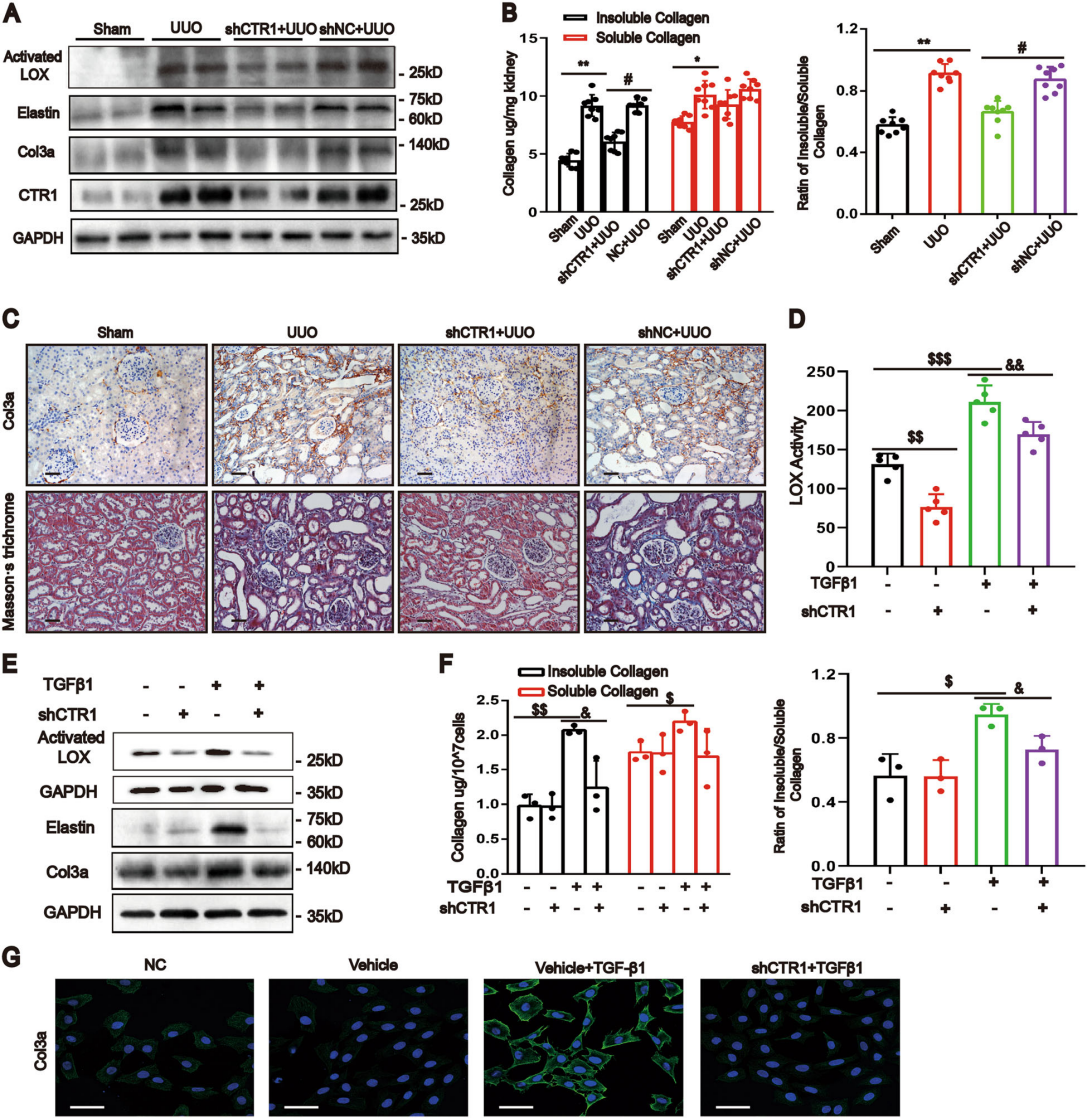
Downregulation of CTR1 attenuates renal fibrosis. **a** Western blotting showing the expression of activated LOX, Elastin, Col3a and CTR1 in the kidneys after UUO or sham operation with delivery of the CTR1 shRNA–expressing plasmid or empty plasmid (*n* = 8). **b** The contents of insoluble and soluble collagen were determined with the microwave-assisted acid digestion method and the proportion of insoluble to soluble collagen were assessed (*n* = 8). **c** Representative micrographs of col3a immunostaining and Masson’s staining of kidney sections (*n* = 8). Original magnification, ×200. Bar = 50 μm. **d** CTR1 knockdown in NRK-49F cells was performed with a lentiviral shRNAmir system. LOX activity as determined using the Fluorometric Lysyl Oxidase Assay Kit in the culture medium of NRK-49F cells (*n* = 5). **e** Western blotting showing the expression of activated LOX, Elastin and Col3a in NRK-49F cells after CTR1 downregulation (*n* = 5). **f** The insoluble collagen, soluble collagen and the ratio of insoluble to soluble collagen were tested in NRK-49F cells (*n* = 3). **g** Immunofluorescence showing Col3a expression (*n* = 3). Original magnification, ×200. Bar = 50 μm. Data represent the mean ± SEM. *P < 0.05, **P < 0.01 versus sham-operated rats. #*P* < 0.05 versus the scramble-treated UUO kidneys. $*P* < 0.05, $$*P* < 0.01, $$$*P* < 0.001 versus the nontreated group. &*P* < 0.05, &&*P* < 0.01 versus with TGFβ1-treated cells.

To confirm the pathogenic role of copper and CTR1 in kidney fibrosis, we knocked down CTR1 by delivering the shRNA plasmid into UUO rats. The results showed that the activated LOX was significantly reduced (Fig. 4a) with lower insoluble collagen and a lower ratio of insoluble to soluble collagen (Fig. 4b) in the CTR1-shRNA-treated kidneys. Meanwhile, the protein upregulation of elastin and collagen 3a (Col3a) was blunted and kidney fibrosis ameliorated in the CTR1-shRNA-treated kidneys (Fig. 4a, c).

In vitro, knockdown of CTR1 blocked the increase in LOX activity (Fig. 4d), the protein levels of activated LOX (Fig. 4e), the insoluble collagen content, the ratio of insoluble/soluble collagen (Fig. 4f) and the protein levels of elastin and Col3a (Fig. 4e, g) induced by TGF-β1. These results indicate that upregulation of CTR1 and copper are responsible for LOX activity enhancement and renal fibrosis.

### Chelating copper ameliorates renal fibrosis

**Fig. 5:**
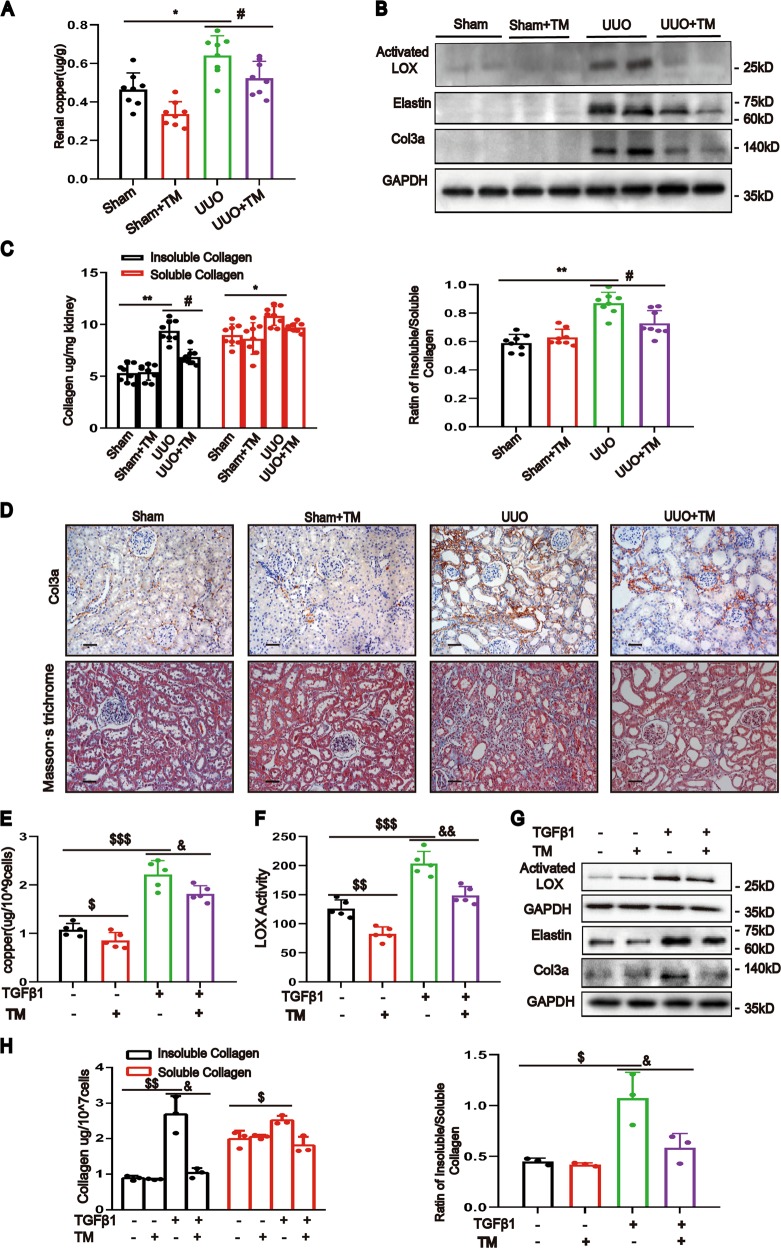
Chelating copper decreased LOX activity and ameliorated renal fibrosis. UUO or sham-operated rats were randomly divided into the following four groups: (1) sham rats treated with PBS; **(**2) sham rats treated with TM (1.2 mg/kg); (3) UUO rats treated with PBS; and (4) UUO rats treated with TM. **a** Copper concentrations of kidneys were determined by ICP-MS (*n* = 8). **b** Western blotting showing actived LOX, elastin and col3a expression in rat kidneys (*n* = 6). **c** The kidney insoluble collagen, soluble collagen and their ratio were determined (*n* = 8). **d** Representative images of col3a immunostaining and Masson’s staining of kidney sections (*n* = 5). Original magnification, ×200. Bar = 50 μm. **e** ICP-MS analysis of copper concentrations in NRK-49F cells at 24 h with or without TM and TGF-β1 treatment (*n* = 5). **f** LOX activity in cell culture medium assay was determined using the Fluorometric Lysyl Oxidase Assay Kit (*n* = 5). **g** Western blotting showing the expression of activated LOX, elastin and col3a (*n* = 3). **h** Microwave-assisted acid digestion showing the insoluble and soluble collagen in NRK-49F cells with or without treatment of TM and the ratio of insoluble to soluble collagen were calculated (*n* = 3). Data represent the mean ± SEM. *P < 0.05, **P < 0.01 versus sham-treated rats. #*P* < 0.05 versus solvent control-treated rats with UUO. $*P* < 0.05, $$*P* < 0.01, $$$*P* < 0.001 versus nontreated cells. &*P* < 0.05, &&P < 0.01 versus TGF-β1-treated cells.

To further confirm the role of copper in renal fibrosis, we used tetrathiomolybdate (TM) to chelate intracellular Cu (II)^16,17^. We found that copper levels decreased after TM treatment (Fig. 5a). This effect was accompanied by decreased activated LOX protein (Fig. 5b), decreased insoluble collagen and a decreased ratio of insoluble/soluble collagen (Fig. 5c). Consistent with these findings, the protein levels of elastin and Col3a in the UUO kidneys decreased, and the fibrosis in UUO kidneys with TM treatment was markedly improved (Fig. 5b, d). In vitro, the TGF-β1-induced increases in copper concentration, LOX activity and LOX protein levels were attenuated upon TM treatment (Fig. 5e–g). In addition, TGF-β1 induced increases in insoluble collagen, the ratio of insoluble/soluble collagen, and the protein levels of elastin and Col3a were markedly attenuated by copper chelation (Fig. 5h, g). These results indicate that the elevated copper influx caused increased LOX activity, facilitating renal fibrosis.

### CTR1 and LOX expression correlated positively with tubulointerstitial fibrosis in the human kidney

**Fig. 6:**
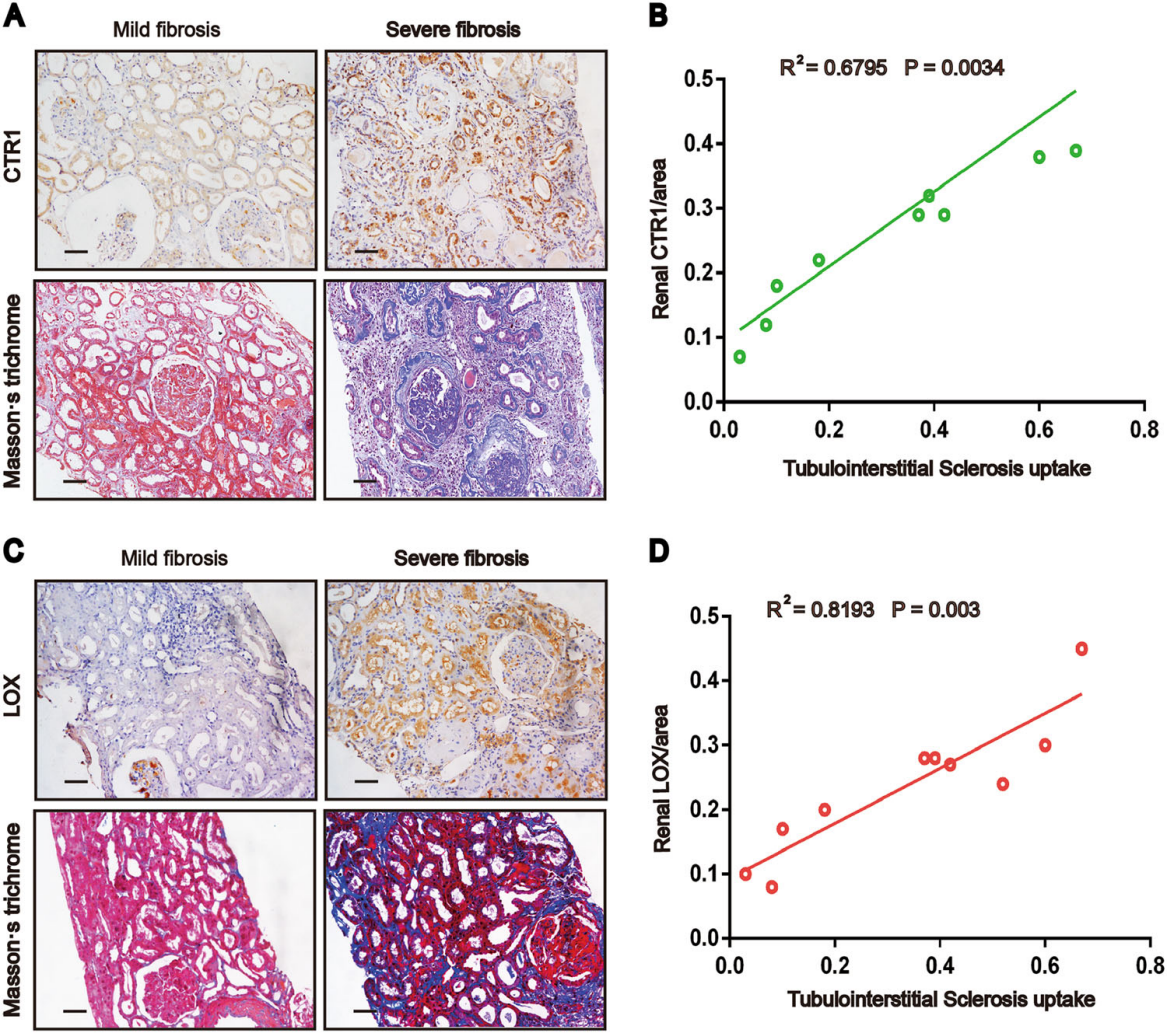
Renal CTR1 and LOX levels correlate with the severity of renal fibrosis in patients. **a**, **c** IHC staining for CTR1 or LOX and Masson’s staining in kidney sections from 10 renal puncture patients. Original magnification, ×200. Bar = 50 μm. **b**, **d** Scatter plots with linear regression showing the correlation between CTR1 expression levels and the extent of renal fibrosis (*R*^2^ = 0.6795, *P* = 0.0034) and the correlation between LOX expression levels and the extent of renal fibrosis (*R*^2^ = 0.8193 *P* = 0.003). The expression of CTR1 and LOX and the level of fibrosis were quantitatively assessed by IHC staining and Masson’s staining, respectively, using a quantitative image-analysis system. Tubulointerstitial fibrosis and LOX and CTR1 expression were quantified based on five randomly selected high-power fields under a microscope equipped with a quantitative image-analysis system (Image-Pro Plus 6.5, Media Cybernetics, Silver Springs, MD), and the results are presented as the percentage of the area with positive staining.

Because the biopsy tissue of patients was limited, we were unable to detect the intrarenal copper concentrations. Therefore, we only tested CTR1 and LOX expression using immunohistochemistry. Kidney tissues were collected from 10 patients with renal biopsy. We found that CTR1 and LOX expression positively correlated with tubulointerstitial fibrosis (Fig. 6a, c). Quantitative analysis of CTR1 staining correlated positively with quantification of the Masson’s trichrome-positive area in the patients (*R*^2^ = 0.6795, *p* = 0.0034) (Fig. 6b). Similar trend was found between LOX expression and renal fibrosis (*R*^2^ = 0.8193, *p* = 0.003) (Fig. 6d).

## Discussion

A major finding in the present study is that the intracellular copper overload was associated with kidney fibrosis both in vivo and in vitro settings. We measured seven metal elements, including copper, zinc, magnesium, manganese, iron, cadmium, and calcium. Among these elements, only copper was clearly elevated in the fibrotic kidney tissue (Fig. 1).

Serum copper concentrations were increased in the two kidney fibrosis models in the present study (Supplementary Fig. 1B). Similarly, several previous studies have reported that increased circulating levels of copper associate with fibrosis and organ damage (liver, lung, oral submucous tissue) in animals^3–5^ and humans^18,19^; however, the mechanism by which an elevated serum copper concentration leading to intracellular copper overload could induce organ fibrosis was not investigated in these studies.

To determine the mechanism of the high tissue copper levels, we focused on copper transport. CTR1 is the most important regulator of the intracellular flow of copper. After CTR1-mediated copper transfer into the cytoplasm, CTR1 shuttles copper to the copper chaperones cytochrome c oxidase copper chaperone (COX17), copper chaperone for superoxide dismutase (CCS) and antioxidant 1 (ATOX1)^20,21^. Previous studies have shown that deletion of CTR1 or cleavage of the CTR1 metal-binding ectodomain in cells and mice lead to reduced intracellular copper levels^22,23^. Another study showed that CHCA-1, as a CTR1 homolog, promotes copper acquisition and affects life activities in C. elegans^24^. In our study, the data showed that the increased intracellular copper influx is vitally controlled by CTR1. TGF-β, a major factor promoting kidney fibrosis, induces Smad2/3 phosphorylation, and phosphorylated Smad2/3 transfer into nucleus and directly bonded to the promoter of CTR1 (Fig. 2). We demonstrated that inhibition of Smad2/3 expression attenuated TGF-β-induced CTR1 expression in NRK-49F cells, indicating the relevance of the Smad2/3 pathway for the activation of CTR1.

The next major finding in our study is that we could demonstrate that an elevated concentration of intracellular copper activates LOX and leads to increased ECM crosslinking that promotes development of kidney fibrosis.

The LOX family is composed of five members (LOX and LOX-like 1 to 4). All LOX family members share two highly conserved domains: a unique copper-binding domain that contains four histidine residues and a cytokine-receptor-like (CRL) domain that is similar to type I cytokine receptors^25,26^. Our study showed that the expression of all LOX genes increased, with the most significant increase in LOX in fibrotic kidney tissue (Fig. 3a, b). We analyzed the structure of LOX in saccharomycetes. There were four direct binding sites for copper ions: His 528, His 530, His 694 and residue 478 of topaquinone^15^. The energies of the LOX-Cu complex were much stronger than the LOX initial state, suggesting that the copper ion in solution can stably bind to LOX and further prevent decomposition of LOX. Therefore, copper is required for LOX’s function and structure. Our results confirmed that the activity of LOX was dependent on the intracellular copper level; copper levels were reduced by CTR1 knockdown (Fig. 4a) and chelating copper could reduce LOX protein activity and stabilization.

We could show that the activity of LOX had a positive relationship with the degree of kidney fibrosis, and that reducing LOX activity would reduce ECM crosslinking and ameliorate kidney fibrosis. We obtained the same results when we used two methods to reduce the intracellular copper: chelating copper with TM and knockdown of CTR. In another study, TM therapy-induced reduction of free copper was also shown to be effective also in ameliorating pulmonary fibrosis induced by bleomycin^27^. In a clinical study of breast cancer, 51 patients received oral TM for 2 years, and both the activity of LOX on collagen crosslinking and the metastases to lungs were reduced^28^. These studies support our findings.

One limitation of the present study is that we did not study the reason for elevated serum copper in the fibrosis model. In other studies of liver fibrosis, elevated serum copper was due to the increased absorption of intestinal copper to the circulation and increased release of copper from damaged cells^29,30^. Another limitation is that we were unable to determine copper content in human kidney tissue to confirm the relationship between copper and fibrosis in CKD patients because we did not have enough kidney tissue from renal biopsy. This indicates the need to develop methods that would make it possible to measure copper from limited human kidney tissue.

**Fig. 7:**
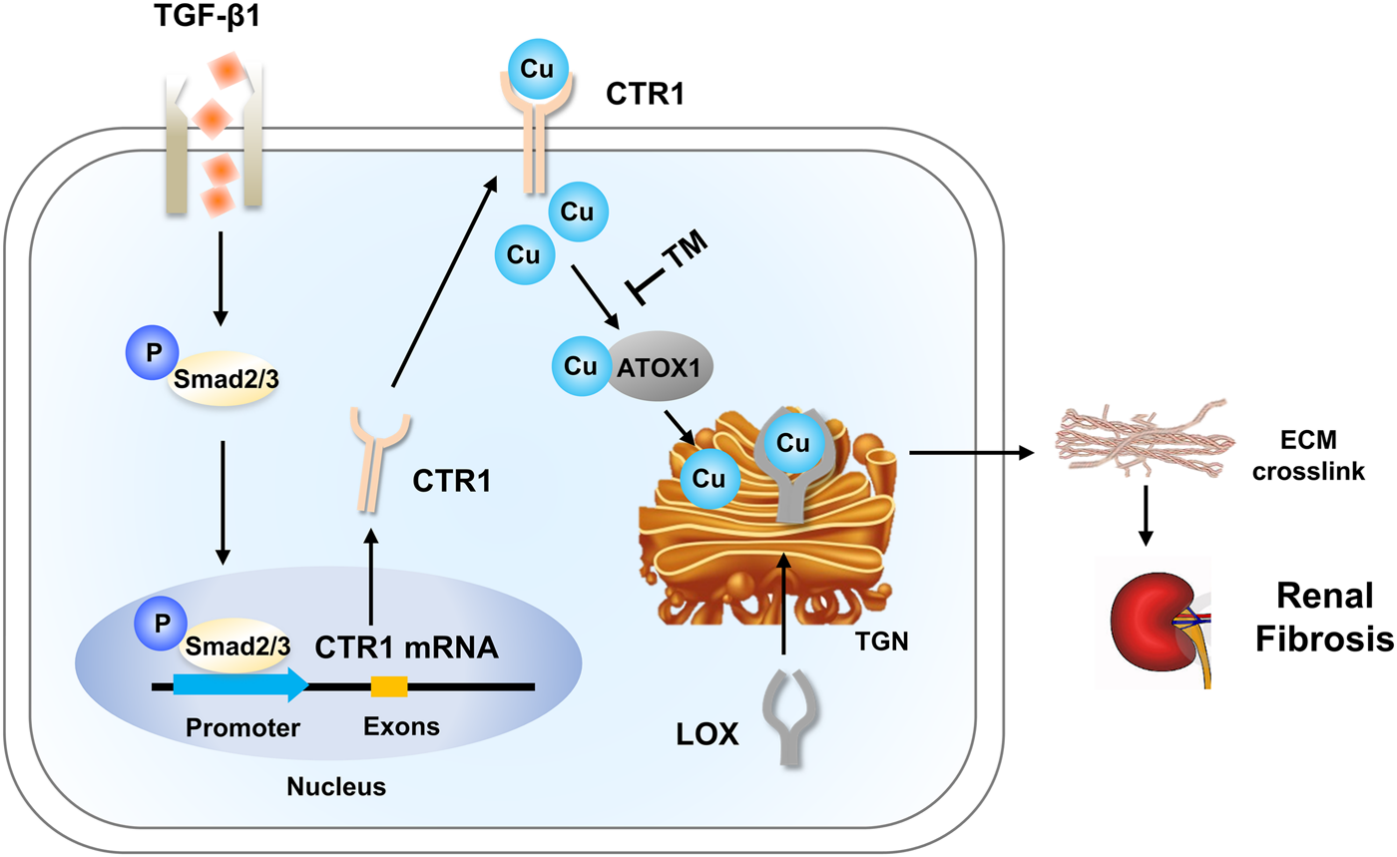
Schematic representation of the novel CTR1-Cu-LOX axis mechanism underlying renal fibrosis. In pathologic conditions, upon TGF-β1 signaling activation, activated pSmad3 translocates to the nucleus, where it binds to the CTR1 promoter to initiate its expression. CTR1 expression induces the influx of copper, which binds, stabilizes and activates LOX. Activated LOX crosslinks ECM, resulting in the acceleration of renal fibrosis.

In conclusion, the present study reveals a novel mechanism underlying renal fibrosis. We report that CTR1-mediated copper transport resulting intracellular copper overload, which is responsible for activation of LOX, which latter increases the crosslinking of collagen and elastin, eventually leading to aggravation of fibrosis in renal tissues (Fig. 7). The results of our study suggest that reduction of intracellular copper by chelating therapy to reduce copper overload might be a novel therapeutic target for prevention and treatment of renal fibrosis.

## Materials and methods

### uIRIx and UUO models

Male Sprague-Dawley (SD) rats (200–250 g) (SLRC Laboratory Animal, Shanghai, China) and 8-week-old male C57Bl/6J mice were used for the in vivo analysis. The use of rats and mice conformed to the National Institutes of Health’s Guide for the Care and Use of Laboratory Animals and was approved by the Ethics Committee of Tongji University School of Medicine. Rats and mice were anesthetized with 2.5% isoflurane.

uIRIx model: C57Bl/6J mice were subjected to uIRIx as previously described^5,31^. Left renal arteries were clamped for 30 min with nontraumatic microaneurysm clamps (Roboz, #RS-6472), followed by reperfusion. The body temperature of the mice was maintained at 37.0 °C. Seven days later, the mice were anesthetized and the right kidney was directly removed. Mice (*n* = 8 per group) were killed at 28 days after ischemia, and kidneys were harvested. Masson’s staining demonstrated moderate blue collagen deposition in uIRIx kidneys.

UUO model: Rats were subjected to unilateral ureteral obstruction (UUO) operation. UUO surgery was performed as previously described^32^. A longitudinal incision parallel to the midline of the abdomen exposes the left kidney and ureter. The left ureter was ligated by surgical 5–0 silk. Rats (*n* = 8 per group) were sacrificed on day 7 after UUO surgery, and the left kidneys were collected. UUO kidneys were marked by prominent fibrosis using Masson’s staining.

### Knock down kidney CTR1 by transporting plasmid in UUO

To deliver the CTR1-shRNA plasmid in vivo, the UUO rats were randomly divided into four groups: (1) sham, (2) UUO, (3) rats treated with CTR1 shRNA before UUO (shCTR1 + UUO), and (4) rats treated with an empty vector before UUO (shNC + UUO). Ultrasound-mediated gene transfer of the CTR1-shRNA technique was executed according to previously described methods^33^. For the shCTR1+UUO and shNC+UUO groups, rats received 200 μl of a solution containing the CTR1 shRNA-pSuper.puro vector or the empty-pSuper.puro vector and lipid microbubbles (Sonovue, Bracco, Milan, Italy) at a ratio of 1:1 (vol/vol) via tail vein injection. Immediately after injection, an ultrasound transducer (MyLab Twice, Esaote Medical, Italy) was directly placed on the skin of the back against the left kidney with a pulse-wave output of 8 MHz at 2 W/cm^2^ for a total of 3 min. UUO was performed immediately, and the rats were sacrificed on day 7.

### Chelating copper with tetrathiomolybdate in UUO

UUO or sham-operated (sham) rats were randomly divided into the following four groups: (1) sham rats treated with PBS by gavage once a day for 7 days after the sham operation; (2) sham rats treated with tetrathiomolybdate (TM) (1.2 mg/kg)^16^ by gavage once a day for 7 days after the sham operation; (3) UUO rats treated with PBS by gavage once a day for 7 days after the UUO operation; and (4) UUO rats treated with TM by gavage once a day for 7 days after the UUO operation. All rats were sacrificed on day 7.

TM, a charge-deficient isosteric polyamine analog, was used to chelate copper in the rats. TM has been used to treat copper toxicosis and Wilson’s disease owing to its efficient intracellular Cu (II)-chelating properties^15^.

### Cell culture

The normal rat kidney fibroblast cell line (NRK-49F), normal rat kidney proximal tubular cells (NRK-52E) and human proximal tubular epithelial cells (HK-2) were cultured in Dulbecco’s modified Eagle’s medium (DMEM) (Gibco, #10569044) supplemented with 10% fetal bovine serum (FBS) (Gibco, #10100147), as described previously^34^. Our results showed the range of copper ion concentration in the culture solution fluctuated at 20–25 μg/L.

### Preparation of a construct expressing CTR1 shRNA

To construct the CTR1 shRNA plasmid, oligos for CTR1 or scramble shRNA were annealed and cloned into the Oligoengine pLVshRNA-mCherry(2A)puro vector using the BamHI sites. To generate a lentivirus, 293T cells were stably transfected with the CTR1 shRNA-pLVshRNA-mCherry(2A)puro vector and a lentiviral vector packaging plasmid mix using Lipofectamine 2000 according to the manufacturer’s instructions (Invitrogen, #11668027). The virus was harvested and transferred into NRK-49F cells. Puromycin was used to screen for cells that were successfully transfected with the lentivirus.

### mRNA sequencing and analysis

Total RNA of kidneys from three rats was extracted with TRIzol (Invitrogen, #15596026). The mRNA was purified from total RNA by an mRNA Purification Kit (Invitrogen, #61006) and was converted to double-stranded cDNA. The cDNA library was generated using the Collibri Stranded RNA Library Prep Kit (Invitrogen, #a38994024). All samples were sent to the GENEWIZ Technology Corporation (SuZhou), and an Illumina platform was used for mRNA sequencing.

The quality of raw sequencing data was assessed with FastQC, which provided a quality profile such as GC content biases, overrepresented sequences, and adapter content. The low-quality reads were then trimmed and filtered by Trimmomatic^35^. The clean reads were mapped to the Rattus norvegicus reference genome (Rnor_6.0) using STAR spliced read aligner^36^, and the Rsubread package in R, which contains the feature Counts function^37^, was applied to determine count matrices of gene counts. The fold change in the gene expression level between the sham and UUO samples was estimated using the GFOLD algorithm^38^.

### Real-time PCR analysis

Total RNA was isolated from cultured cells or kidney tissues using TRIzol (Invitrogen, #15596026), as previously described^34^. The primers used in this study included rat GAPDH, CTR1, LOX, LOXL1–4, Smad2, and Smad3 (Table 1).

**Table 1:** Sequences of the primer pairs used for real-time PCR.

Name	Forward Primer	Reverse Primer
GAPDH	5′ GGCAAGTTCAACGGCACA 3′	5′ CCATTTGATGTTAGCGGGAT 3′
CTR1	5′ GTATTTGGTGGCTGGGGTTC 3′	5′ TCGGGTTTCGGCGTAAGT 3′
LOX	5′CATTACCACAGCATGGATGAATTC 3′	5′CAGTCTATGTCTGCTGCATAAGTA 3′
LOXL1	5′ TACTTGCCTGTGCGAAACTCT 3′	5′ GTGGATGCCTGCACGTAGTT 3′
LOXL2	5′ GCATGGATTTGGCATGACTG 3′	5′ GCACACTCGTAACTCTTCTG 3′
LOXL3	5′ CCTCTTTATGCCACCTCTAC 3′	5′ GCTGCCTGCAGATCCCAC 3′
LOXL4	5′ TGGATTCACCGGGTTGACTG 3′	5′ GATGGTCTGCTCATAGCCCC 3′
Smad2	5′ GGGAAGTGTTTGCCGAGTG 3′	5′ CTGCTGACGGACTTTAGGACA 3′
Smad3	5′ TGCCAGCGATGGACTTTATG 3′	5′ CACGACTGCCCTTGTTGC 3′

### Western blot analysis

Kidney tissue and cell lysates were prepared using radioimmunoprecipitation assay (RIPA) buffer. Samples were run on 10% SDS-PAGE gels and transferred to PVDF membranes. The membranes were incubated with primary antibodies against elastin, Col3a (Abcam, #ab-21610 and #ab-6310, respectively) and GAPDH (Santa Cruz, #sc-137179), followed by incubation with the secondary antibody (CST, #5127) conjugated to horseradish peroxidase. Signals were detected using an Odyssey Infrared Imaging System. Densitometry was performed using Quantity One software.

### Immunostaining analysis

Staining was performed on 2-μm-thick paraffin kidney sections that were pretreated with a heat-based antigen retrieval approach. The primary antibodies used were Col3a (Abcam, #ab-6310), LOX (Abcam, #ab-60178), α-SMA (a specific marker of myofibroblasts) (Abcam, #ab-7817), Pan Cytokeratin (a specific marker of epithelial cell) (Abcam, #ab-7753) and CTR1 (Abcam, #ab-129067). The sections were incubated with the primary antibodies at 4 °C for 1–2 nights, followed by incubation with biotin-labeled secondary antibodies and horseradish peroxidase (HRP)-labeled tertiary antibodies (DAKO). The sections were developed using a 3,3′-diaminobenzidine (DAB) kit (Vector, #SK4100), counterstained with hematoxylin, dehydrated, and examined under a light microscope. For colocalization immunofluorescence staining, the slides were incubated with Alexa Fluor conjugated secondary antibody (Invitrogen, #Alexa Fluor 568-A11011, Alexa Fluor 488- A11029) for 2 h at room temperature. Zeiss confocal microscope was used to observe the labeled slides.

### Chromatin immunoprecipitation analysis

ChIP was performed using a Transcription Factor ChIP kit according to the manufacturer’s instructions. NRK-49F cells were treated with or without TGF-β1 for the indicated times. Cells were then crosslinked with 1% formaldehyde for 10 min at 37 °C and quenched with glycine, followed by sonication using a Bioruptor (Diagenode, Liege, Belgium) to generate 150- to 600-bp DNA fragments. Immunoprecipitation was performed with an antibody against Smad3 (CST, #9513), and normal IgG was used as a control. Precipitated DNA samples were examined by PCR for the presence of the CTR1 promoter sequence using the following primers: forward, 5′ GGACGACAACATCACCAT 3′; reverse, 5′ CCACATTCTTAAAGCCAAA 3′.

### Inductively coupled plasma-mass spectrometry

Samples were collected and stored in metal-free plastic collection tubes for ICP-MS. The samples were dissolved in concentrated nitric acid and then heated in a high-temperature resolution stove. Then, all samples were diluted with purified water. Standard samples were purchased (Sero, #1103129) and used for quantification. The concentration of copper was quantified by ICP-MS on a NexION instrument (Fudan University, Shanghai, China) and analyzed using argon^39,40^.

### LOX activity

LOX activity was measured using the Fluorometric Lysyl Oxidase Assay Kit (AAT Bioquest, #15255) in conditioned medium from the NRK-49F cell culture. The conditioned medium was prepared using phenol red-free DMEM, and the NRK-49F cells were cultured for 24 h with and without TM treatment. Phenol red-free DMEM that was not incubated with the cells was used as a blank control. LOX activity was determined according to the manufacturer’s instructions by monitoring and measuring LOX-catalyzed H_2_O_2_ release from the fluorescent substrate in an HRP-coupled reaction.

### Collagen crosslinking

Kidney collagen was fractionated into pepsin-soluble and pepsin-insoluble parts, with the insoluble collagen being largely crosslinked^41^. The renal tissue was dissociated by incubation in 0.5 mol/L acetic acid for 12 h and then treated with 5 mg/ml pepsin for 12 h. Pepsin-soluble and pepsin-insoluble collagens were separated by centrifugation at 3000 *×* *g* for 10 min at 4 °C. The two collagen fractions were hydrolyzed by a high-pressure tank and then quantified by measuring hydroxyproline using the modified Stegemann method^42,43^.

### Renal biopsy collection

To examine CTR1 and LOX expression, we collected renal tissues from 10 renal puncture patients at Tongji Hospital from 1 January 2018, to 30 June 2018. The 10 patients included 3 patients with minimal-change disease, 1 patient with IgA nephropathy, 3 patients with diabetic nephropathy, 2 patients with membranous nephropathy, and 1 patient with glomerulosclerosis. The features of demographic, clinical and pathology of these patients are listed in Table 2. Masson’s trichrome staining and immunohistochemical staining were used. Two independent pathologists scored the renal fibrosis specimens. The protocol for the use of patient samples in this study was approved by the Human Subjects Committee of Tongji Hospital (Clinical trial registration number: Lunwei-KYSB-2016-103). Informed consent was obtained from all participants.

**Table 2:** Demographic and clinical feature of the renal puncture patients.

Variable	Renal puncture patients (*n* = 10)
*Age, years*	
Mean ± s.d.	62.9 ± 9.4
Median (range)	62 (41–73)
*Sex, no (%)*	
Male	7 (70%)
Female	3 (30%)
*Smoking status, no. (%)*	
Ever and current	5 (50%)
Never	5 (50%)
*Alcohol consumption, no. (%)*	
Ever and current	4 (40%)
Never	6 (60%)
*Histological types, no. (%)*	
Minimal-change disease	3 (30%)
IgA nephropathy	1 (10%)
Diabetic nephropathy	3 (30%)
Membranous nephropathy	2 (20%)
Glomerulosclerosis	1 (10%)
*Renal function*	
Creatinine Mean ± s.d.	158.1 ± 79.5
Blood Urea Nitrogen Mean ± s.d.	12.624 ± 7.8
*Fibrosis area*	
Mean ± s.d.	33.6% ± 21.6%
Median (range)	37% (3–67%)

### Ab initio calculations

Ab initio calculations based on density functional theory (DFT) were implemented in the Gaussian09 package^44^. The geometry optimizations were carried out at the DFT level, employing the M06L functional^45^. A mixed basis set GEN (SDD basis sets for copper atom, and 6–31 + G (d,p) set for other atoms) was applied for all the calculations in this study. The optimized stationary points were identified as minima or first-order saddle points. Solvation effects of the outer water environment were taken into account by calculating the single-point energies of the optimized configurations under the integral-equation-formalism polarizable continuum model (IEFPCM) of solvation^46^ at the same level of theory as that used in the gas-phase optimizations.

### Statistical analysis

All values examined are shown as the mean ± SEM for each group. Intergroup comparisons were made using one-way analysis of variance. Student’s *t*-test was used to analyze data between two groups. All experiments were repeated at least three times. In all tests, *P* < 0.05 was considered a statistically significant difference between the mean values. SPSS 21.0 software (Chicago, IL) was used for the statistical analyses.

## Supplementary information

Supplementary legends

Supplement figure1

Supplement figure2

Supplement figure3

## Data Availability

Upon reasonable request, the data that support the results of this study can be obtained from the corresponding author.
