# Supplementary material for: Elevated intracellular copper contributes a unique role to kidney fibrosis by lysyl oxidase mediated matrix crosslinking
Source: Cell Death Dis. 2020 Mar 31;11(3):211. doi: 10.1038/s41419-020-2404-5 (PMC7109154; doi:10.1038/s41419-020-2404-5)
Supplement: Supplementary file 1 — Supplementary legends [file 41419_2020_2404_MOESM1_ESM.docx]

**Supplementary legends**

**Supplementary Figure 1. Copper ion and CTR1 expression were elevated in NRK-49F and NRK-52E cells and UUO rat serum and ultrasound-mediated gene transfer of a plasmid expressing CTR1 shRNA in the UUO kidney**

(A) ICP-MS analysis showing the copper concentrations in NRK-52E cells at 48 h with or without TGF-β1 treatment (n=5). (B) The serum copper concentrations in UUO and sham rats were tested by ICP-MS (n=6). (C) Renal images at different time points after ultrasound treatment, including (a) 7 s, (b) 28 s, (c) 1 min 18 s, and (d) 2 min 39 s following the injection of plasmid DNA with lipid microbubbles via tail vein injection. (D, E) Western blotting for CTR1 in NRK-52E and HK-2 cells at 24 h stimulated by TGF-β1 (n=3).

Data represent the mean±SEM. **P<0.01 versus nontreated cells. ##P<0.01 versus sham-treated rats.

**Supplementary Figure 2. Analysis of the generation of a stably transfected NRK-49F cell line expressing a CTR1 shRNA** **with the pLVshRNA-mCherry(2A)puro plasmid and simultaneous knockdown of Smad2 and Smad3 using siRNA in NRK-49F cells**

(A) Fluorescence and light microscopy examination of cells treated separately with two independent CTR1 shRNAs (n=3). Original magnification, 200X. Bar = 50 μm. (B, C) Western blotting and real-time PCR showing CTR1 expression in NRK-49F cells transfected with a scramble control plasmid or two CTR1 shRNA plasmids (n=3). (D, E) Western blotting and real-time PCR for Smad2 and Smad3 by the siRNAs in NRK-49F cells (n=3).

Data represent the mean±SEM. **P<0.01, ***P<0.001 versus scramble shRNA-treated cells. ##P<0.01, ###P<0.001 versus scramble shRNA-treated cells.

**Supplementary Figure 3. Ultrasound-mediated gene transfer of a plasmid expressing CTR1 shRNA in the normal kidney**

To confirm the role of copper and CTR1 in normal kidney, we knocked down CTR1 by delivering the shRNA plasmid into normal rats. Rats were randomly divided into normal group, shCTR1-3days group and shCTR1-7days group and the level of renal copper, serum creatinine, blood urea nitrogen, kidney Col3a, kidney elastin, renal soluble collagen and renal insoluble collagen were tested respectively. (A) ICP-MS analysis showing the copper concentrations in rat kidney(n=3). (B) The levels of serum creatinine were measured by colometric methods (n=3). (C) Serum level of blood urea nitrogen after knocking down CTR1(n=3). (D)Western blotting showing the expression of elastin and col3a (n=3). (E) The insoluble, soluble collagen and the ratio of insoluble to soluble collagen were assessed in the rat kidneys (n=3).

Data represent the mean±SEM. **P<0.01 versus normal group.
