# Supplementary figures and images for: Elevated intracellular copper contributes a unique role to kidney fibrosis by lysyl oxidase mediated matrix crosslinking
Source: Cell Death Dis. 2020 Mar 31;11(3):211. doi: 10.1038/s41419-020-2404-5 (PMC7109154; doi:10.1038/s41419-020-2404-5)

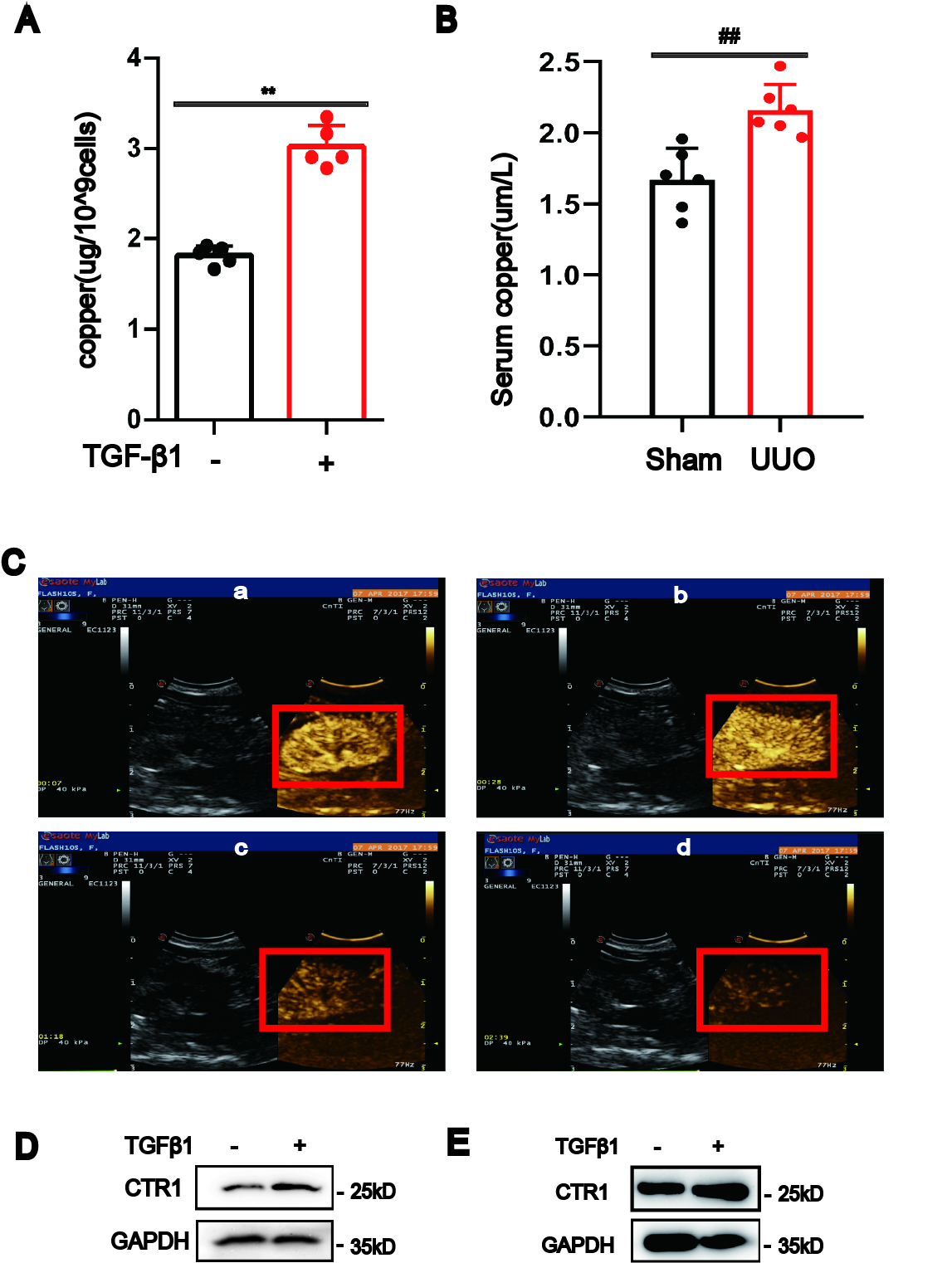

Supplement: Supplementary file 2 — Supplement figure1 [file 41419_2020_2404_MOESM2_ESM.tif]

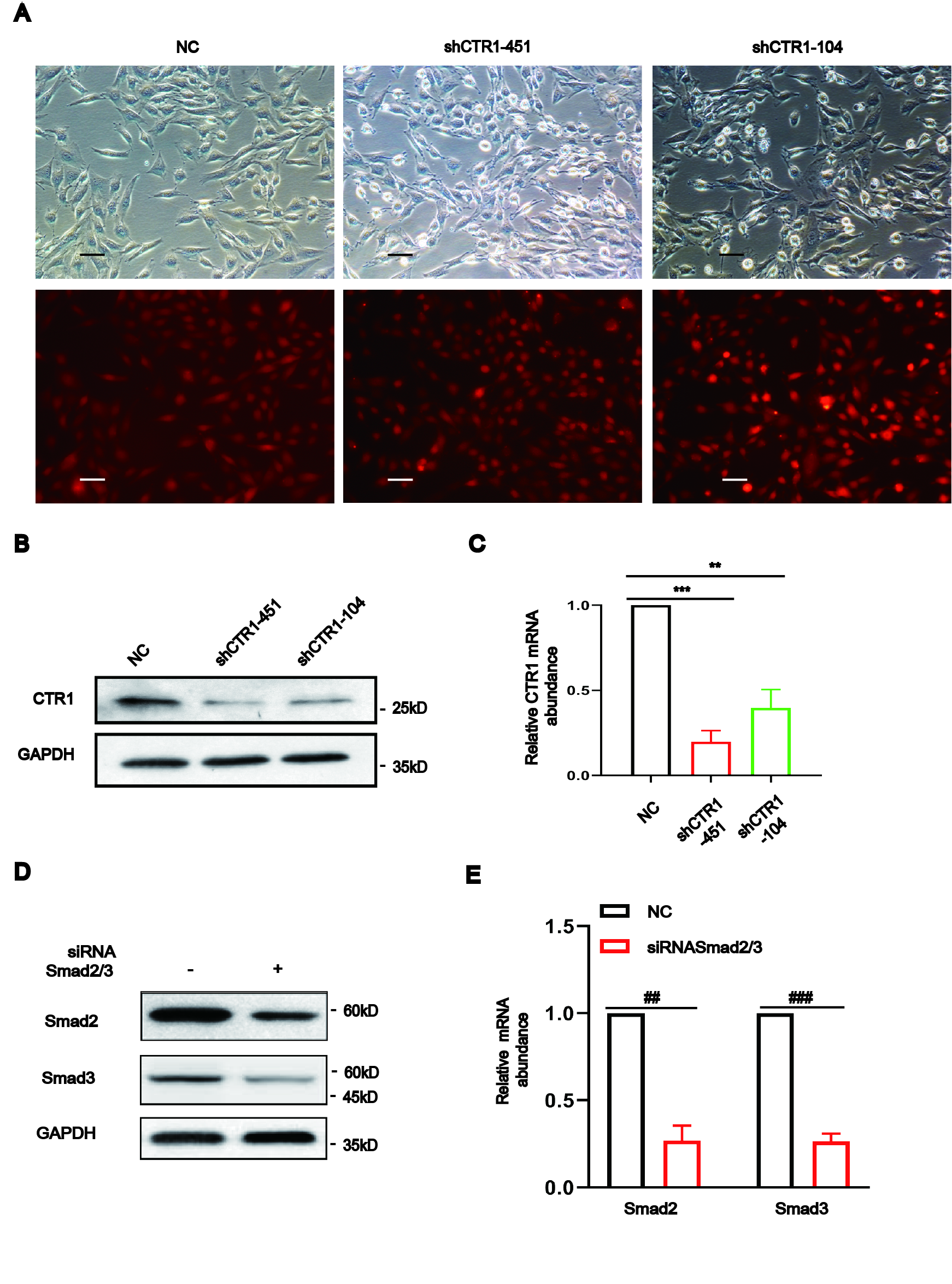

Supplement: Supplementary file 3 — Supplement figure2 [file 41419_2020_2404_MOESM3_ESM.tif]

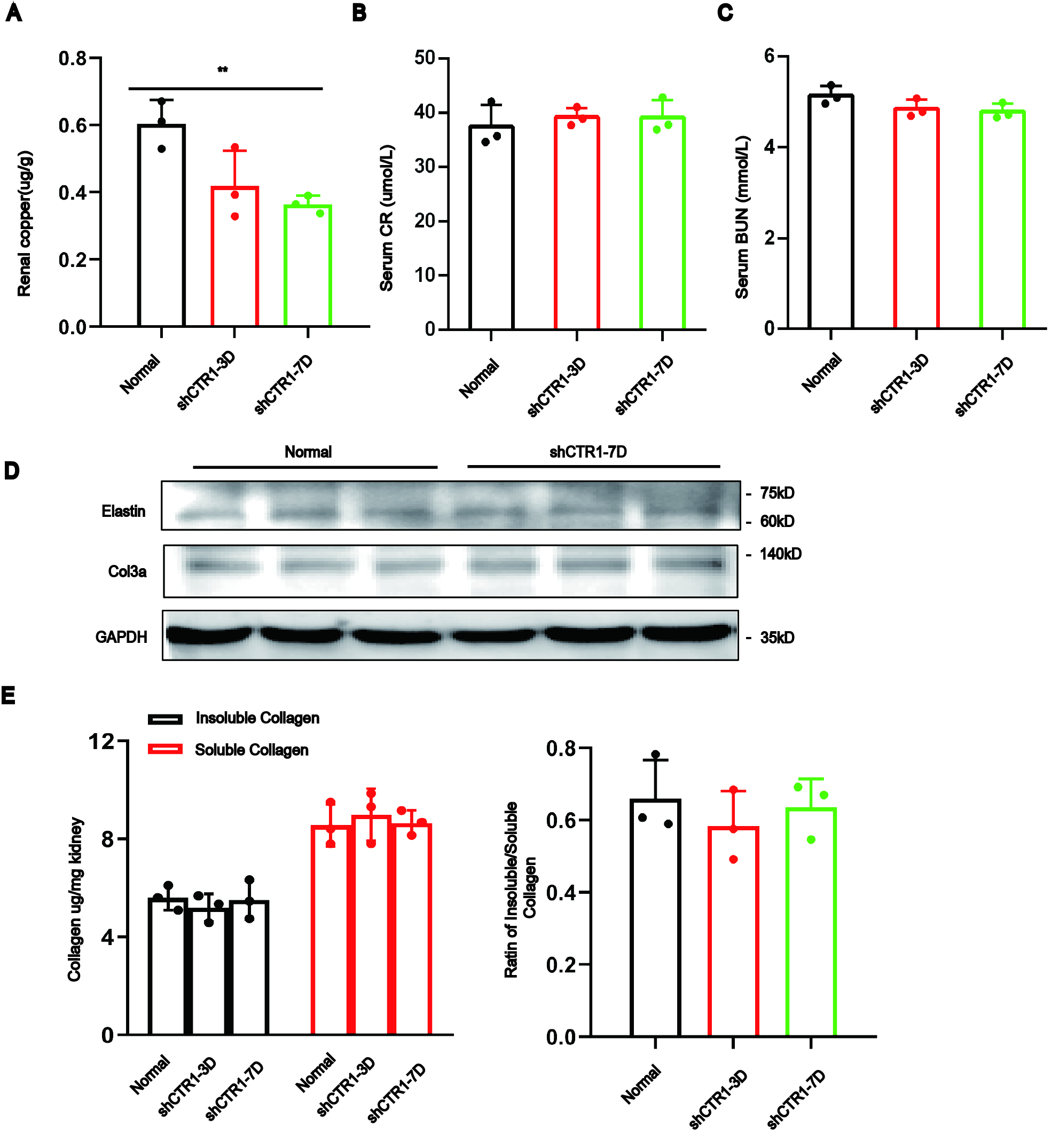

Supplement: Supplementary file 4 — Supplement figure3 [file 41419_2020_2404_MOESM4_ESM.tif]
